# Two rare presentations of embryonal rhabdomyosarcoma of the cervix in teenagers at a low-resource teaching hospital in Ghana: A case series

**DOI:** 10.1016/j.gore.2021.100750

**Published:** 2021-03-19

**Authors:** Sarah G. Bell, Thomas Okpoti Konney, Adu Appiah-Kubi, Augustine Tawiah, Kwabena Amo-Antwi, John Jude Kweku Annan, Emma R. Lawrence, Richard Lieberman, Carolyn Johnston

**Affiliations:** a University of Michigan, Department of Obstetrics and Gynecology, 1500 E. Medical Center Dr., Ann Arbor, MI 48109, USA; b Komfo Anokye Teaching Hospital, Okomfo Anokye Road, Kumasi, Ghana

**Keywords:** Embryonal rhabdomyosarcoma, Sarcoma botryoides, Fertility-sparing surgery

## Abstract

We report two cases of embryonal rhabdomyosarcoma (ERMS) of the cervix in teenagers presenting to Komfo Anokye Teaching Hospital in Kumasi, Ghana within one month of each other. Between October and November 2019, two patients presented with ERMS of the cervix. They both underwent fertility-sparing surgery followed by chemotherapy with vincristine, actinomycin-D, and cyclophosphamide. Preoperative workup for the two patients was minimal due to limited availability and high cost of imaging in a low-resource setting. Both patients were discussed at a multidisciplinary tumor board meeting to guide best management practices. Both patients had local surgical resection with histological confirmation of ERMS and negative margins, followed by six cycles of vincristine, actinomycin-D, and cyclophosphamide. Neither of the patients had perioperative complications or received radiation therapy. At the time of publication, both patients are currently alive and without evidence of recurrence. Fertility-sparing surgery followed by chemotherapy for patients with ERMS of the cervix is accessible in low-income countries.

## Highlights

• We report on two cases of embryonal rhabdomyosarcoma of the cervix in Ghana.
• Only 20% of rhabdomyosarcoma diagnoses in children occur in the genitourinary tract.
• Embryonal rhabdomyosarcoma of the cervix is rare, with no standardized treatment.
• The teenaged patients underwent fertility-sparing surgery followed by chemotherapy.
• This treatment regimen is accessible in low-income countries.

## 1 Introduction

Rhabdomyosarcoma (RMS) is the most common soft tissue tumor of children. RMS primarily arises in the head and neck region or the genitourinary tract. There are three major subtypes of RMS: embryonal, alveolar, and undifferentiated (Parham and Barr, 2013). Embryonal rhabdomyosarcoma (ERMS) is the most common subtype and can be further classified into the classic subtype, botryoid subtype, and spindle cell subtype (Kriseman et al., 2012). It is estimated that 20% of RMS cases in children arise in the genitourinary tract and only 0.5% of these tumors arise from the cervix (Arndt et al., 2001). RMS of the female lower genital tract typically presents in different decades of life, with vaginal RMS typically presenting in the first few years of life, cervical RMS in the second decade, and uterine fundus RMS in postmenopausal women (Arndt et al., 2001).

The ERMS subtype known as sarcoma botryoides typically presents with vaginal bleeding and a polypoid mass protruding from the vaginal introitus. Since cervical ERMS is extremely rare, there is no standardized treatment, but most patients receive a combination of surgical resection and chemotherapy. Surgical treatment has shifted over the past several decades from radical exenterations to local surgical resection in appropriate candidates (Harel et al., 2016).

Based on several studies performed by the Intergroup Rhabdomyosarcoma Study Group, the five-year survival among patients with localized disease was not statistically different among those who did versus those who did not undergo postoperative radiation treatment. These findings shifted treatment to allow patients the opportunity to maintain fertility without undergoing radiation therapy. Instead, radiation therapy is primarily reserved as salvage therapy for those suffering from recurrence or extensive disease, and for those who may not tolerate chemotherapy (Kriseman et al., 2012).

Overall, ERMS of the cervix is exceedingly rare and there are few case reports that outline management and treatment for the disease, especially in resource-limited settings. This case series aims to fill this gap by reporting on two patients with ERMS of the botryoid subtype and to discuss the process of diagnosis and management in a low-resource setting.

## 2 Methods

We report on two cases of embryonal RMS of the botryoid subtype of the cervix at a tertiary hospital in Kumasi, Ghana who presented within one month of each other. Both patients were offered fertility-sparing surgery with local surgical resection followed by chemotherapy. The first patient had a preoperative workup including examination under anesthesia to determine the extent of the disease. The second patient had a preoperative workup with a chest x-ray, as well as a complete blood count, liver panel, and renal panel.

## 3 Results

### 3.1 Case #1

**Fig. 1:**
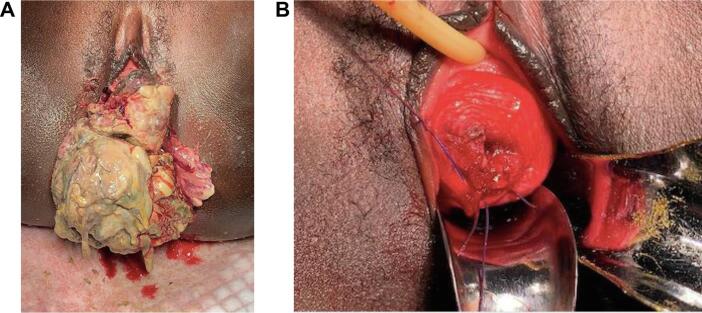
**Preoperative and postoperative images for Case #1.** A. Preoperative. B. Postoperative.

The first patient was a 17-year-old gravida zero who was referred due to a one-year history of an enlarging mass protruding from her introitus associated with vaginal bleeding. She had initially presented five months prior, at which time a biopsy was obtained that confirmed ERMS. However, she was lost to follow-up until she represented after several months with worsening pain and excessive growth of the mass. On subsequent examination under general anesthesia, the mass was found to be protruding from the posterior cervix from 3 o’clock to 9 o’clock. The cervix measured six centimeters in the widest diameter and had a clear line of demarcation separating it from the fibrotic, necrotic mass. The mass was free from her vulva, vaginal walls, and parametria. She underwent polypectomy of the mass using electrocautery and suture. There was no sampling of the endocervix or endometrium at the time of the surgery. Histopathology from Komfo Anoyke Teaching Hospital was again consistent with ERMS botryoides. The final pathologic margins were negative. Per the medical oncology local protocols at KATH, she completed six cycles of vincristine, actinomycin-D, and cyclophosphamide. Fig. 1 shows the preoperative and postoperative images for this patient.

### 3.2 Case #2 (Fig. 2)

The second patient was a 16-year-old gravida zero who was referred due to a grape-like mass protruding from her introitus and acute urinary retention. She first noticed the mass 12 months prior, at which time it was biopsied and confirmed to be ERMS botryoides. Unfortunately, due to lack of transportation and access to a form of communication, the patient was lost to follow-up for 12 months. On physical examination, her abdomen was full, soft, and tender in the suprapubic region. On pelvic examination, she had a polypoid mass measuring 7 × 12 cm protruding from her introitus that bled easily with contact. She had a hemoglobin of 9.3 g/dL with normal liver and renal function tests and normal chest x-ray. Her pelvic ultrasound revealed a bulky uterus with an ill-defined heterogeneous mass, with areas of cystic degeneration in the body of the uterus and cervix. On examination under anesthesia, the mass was 20 × 24 cm, polypoid and smooth, and involved the top and bottom lip of her cervix. She had a grossly normal appearing vulva and vagina, and the parametria were free. The uterus measured an approximate 10-week size.

**Fig. 2:**
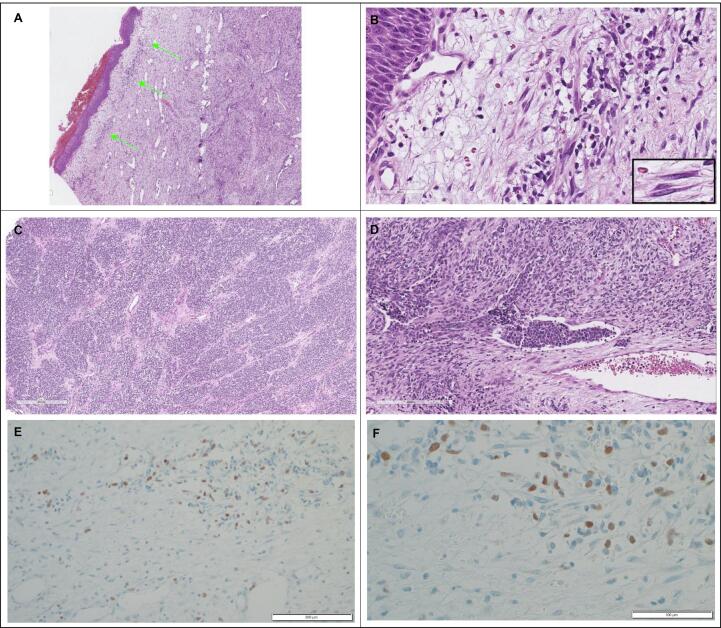
**Pathology slides for Case #2.** A. Sarcoma botryoides (4x): The cambium layer (green arrows) is represented by a subepithelial layer of the spindle cell neoplasm. B. Sarcoma botryoides (14x): Higher power review of the cambium layer. On high-power, the spindled cells can sometimes demonstrate the cross striations of skeletal muscle (INSET: rhabodomyoblasts). C. Alveolar rhabdomyosarcoma (6x): A significant component of this patient’s very large tumor demonstrated a nested pattern of the small round blue cell tumor, also referred to as “alveolar” differentiation of a rhabdomyosarcoma, or alveolar rhabdomyosarcoma. D. Sarcoma botryoides (17x): This portion of the tumor demonstrates a vascular extension (LVI) of the small round blue cell (alveolar) component of the tumor in a background of spindle cell differentiation. E. Myogenin (20x): Microphotography showing nuclear positivity for myogenin. F. Myogenin (40x): Higher power view of microphotography showing nuclear positivity for myogenin. (For interpretation of the references to colour in this figure legend, the reader is referred to the web version of this article.)

**Fig. 3:**
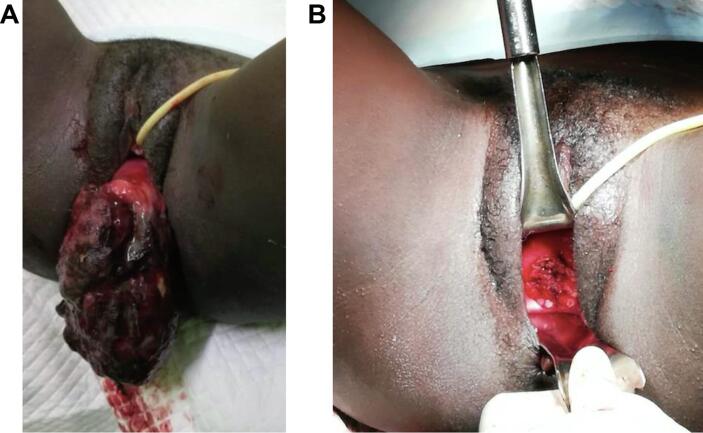
**Preoperative and postoperative images for Case #2.** A. Preoperative. B. Postoperative.

She underwent local resection of the mass three days after initial presentation. The final pathologic margins were confirmed negative at the University of Michigan following discussions at an international multidisciplinary tumor board meeting. Histopathology revealed a spindle cell tumor composed of a biphasic spindle cell neoplasm composed of strap cells (skeletal muscle) and eosinophilic rounded cells (basket cells). A cambium layer—a subepithelial layer of rhabdomyoblasts commonly associated with sarcoma botryoides—was also noted (Fig. 2A and 2B). More undifferentiated areas with features of alveolar rhabdomyosarcoma were also noted, with nests of small round blue cells with brisk mitotic activity, vesicular nuclei, islands of cartilaginous differentiation, extensive tumor necrosis, and readily apparent vascular space invasion (Fig. 2C and 2D). Immunohistochemically, nuclear positivity for myogenin is readily apparent in both variations of this aggressive tumor (Fig. 2E and 2F). Per the medical oncology protocols at KATH, she completed six cycles of vincristine, actinomycin-D, and cyclophosphamide. Fig. 3 shows the preoperative and postoperative images for this patient.

## 4 Discussion

Since ERMS of the cervix is an exceedingly rare disease, it often follows the treatment guidelines for genitourinary primary embryonal RMS. A 2001 study of RMS of the female genital tract demonstrated overall survival of 82%. Patients with the RMS of the botryoid subtype had the highest five-year survival at 94% (Arndt et al., 2001). Over the past several decades, literature suggests that certain patients with RMS of the cervix may be candidates for local resection followed by chemotherapy. This allows women to maintain fertility without undergoing hysterectomy or radiation therapy (Kriseman et al., 2012). After resection, these patients should undergo adjuvant chemotherapy with vincristine, actinomycin D, and cyclophosphamide (Raney et al., 2011). Both of our patients underwent primary surgical management due to their symptomatic masses, with the hope that they would be candidates for chemotherapy after local resection. Although the ideal conservative surgical procedure has not yet been identified, these two cases demonstrated successful management with local resection via polypectomy and conization with negative margins followed by chemotherapy for fertility preservation. Since it is not considered fertility-sparing, radiation therapy should be reserved for instances of recurrence and for treatment of gross residual disease following surgery or chemotherapy.

An important finding in several case reports is the frequency with which patients were noted to have a second primary malignancy in addition to embryonal RMS of the cervix— including at least seven patients with a Sertoli-Leydig tumor of the ovary detected either before or after diagnosis of embryonal RMS of the cervix (Daya and Scully, 1988, Dehner et al., 2012, Golbang et al., 1997, Kriseman et al., 2012, McClean et al., 2007). RMS is known to be associated with several inherited genetic diseases such as Li-Fraumeni syndrome, neurofibromatosis type I, Costello syndrome, Beckwith-Wiedemann syndrome, Noonan syndrome, and multiple endocrine neoplasia type 2A (Huh and Skapek, 2010, Jones et al., 2010, Jongmans et al., 2011, Kriseman et al., 2012, Quezada and Gripp, 2007, Smith et al., 2001). Genetic counseling and testing is not financially available for our patients at this time, but could be considered in the future if accessible.

These two cases add to the growing literature of ERMS of the cervix. A recent case series was published of three patients who underwent fertility-sparing surgeries and chemotherapy without evidence of recurrence at initial post-treatment visits (Bouchard-Fortier et al., 2016). Although our two patients had large tumors, the surgical margins were negative, they were both disease-free at their initial and subsequent postoperative visits, which are occurring every three months since completion of chemotherapy, and both are currently alive at the time of this publication. We recognize that our study is limited by the short interval since our patients’ initial presentation. The long-term management of these patients and others like them is important to determine the best up-front treatment, as well as potential treatment for recurrence.

Notably, both of these cases were managed in a tertiary facility in a low-resource setting, which added additional limitations and complexity to their management. Financial accessibility to imaging and chemotherapy is limited in low-resource settings. At KATH, chemotherapy for pediatric patients is often funded by private foundation donations and thus was available for our two patients. However, imaging for initial staging was not affordable for the two patients and their families.

## 5 Conclusions

Fertility-sparing surgery followed by chemotherapy for patients with ERMS of the cervix is accessible in low-income countries. Since it is not considered fertility-sparing, radiation therapy should be reserved for instances of recurrence and for treatment of gross residual disease following surgery or chemotherapy.

## Ethical approval and patient consent

The institutional review boards at both institutions approved this study (KATH: KATH-IRB/AP/131/20; University of Michigan: HUM00189244, not regulated). Both patients gave verbal consent for their clinical details and photos to be shared, following the standard approach at Komfo Anokye Teaching Hospital for verbal rather than written consent given low patient literacy.

## CRediT authorship contribution statement

**Sarah G. Bell:** Conceptualization, Data curation, Investigation, Project administration, Resources, Validation, Writing - original draft, Writing - review & editing. **Thomas Okpoti Konney:** Conceptualization, Supervision, Writing - review & editing. **Adu Appiah-Kubi:** Conceptualization, Data curation, Investigation, Writing - review & editing. **Augustine Tawiah:** Conceptualization, Data curation, Investigation, Writing - review & editing. **Kwabena Amo Antwi:** Data curation. **John Jude Kwaku Annan:** Data curation. **Emma R. Lawrence:** Conceptualization, Data curation, Investigation, Writing - review & editing. **Richard Lieberman:** Software, Supervision. **Carolyn Johnston:** Supervision, Validation, Writing - review & editing.

## Declaration of Competing Interest

The authors declare that they have no known competing financial interests or personal relationships that could have appeared to influence the work reported in this paper.
